# Autism spectrum disorder detection from semi-structured and unstructured medical data

**DOI:** 10.1186/s13637-017-0057-1

**Published:** 2017-02-01

**Authors:** Jianbo Yuan, Chester Holtz, Tristram Smith, Jiebo Luo

**Affiliations:** 1 Department of Computer Science, University of Rochester, Rochester, 14627 NY USA; 2 School of Medicine and Dentistry, University of Rochester Medical Center, Rochester, 14642 NY USA

**Keywords:** Autism spectrum disorder, Distributed representation, Medical forms, Classification

## Abstract

Autism spectrum disorder (ASD) is a developmental disorder that significantly impairs patients’ ability to perform normal social interaction and communication. Moreover, the diagnosis procedure of ASD is highly time-consuming, labor-intensive, and requires extensive expertise. Although there exists no known cure for ASD, there is consensus among clinicians regarding the importance of early intervention for the recovery of ASD patients. Therefore, to benefit autism patients by enhancing their access to treatments such as early intervention, we aim to develop a robust machine learning-based system for autism detection by using Natural Language Processing techniques based on information extracted from medical forms of potential ASD patients. Our detecting framework involves converting semi-structured and unstructured medical forms into digital format, preprocessing, learning document representation, and finally, classification. Testing results are evaluated against the ground truth set by expert clinicians and the proposed system achieve a 83.4% accuracy and 91.1% recall, which is very promising. The proposed ASD detection framework could significantly simplify and shorten the procedure of ASD diagnosis.

## Introduction

Autism spectrum disorder (ASD) is a general classification for a broad range of disorders with a variety of issues stemming from complications with neurological development. Symptoms of ASD are of varied severity involving difficulties with verbal and nonverbal communication, repetitive behaviors, and typical social interaction. The defining features of ASD are deficits in reciprocal social communication and frequent or intense repetitive or restrictive behaviors [1]. ASD has a prenatal or early childhood onset and a chronic course. Although previously considered rare, ASD is now estimated to occur in approximately 1 in 68 individuals, a threefold increase reported in prevalence in 10 years [2]. No conclusions have been reached on whether the rising prevalence estimates reflect an actual increase in prevalence or are just an artifact of changes in screening and diagnosis. Studies also show that ASD is about four times more common among boys than girls.

Currently, no laboratory test for ASD exists, and the process of diagnosing the disorder is highly complex and labor-intensive, requiring extensive expertise. Clinicians diagnose ASD based on a variety of factors including a review of medical records, medical and neurological examinations, standardized developmental tests, and behavioral assessments, such as the Autism Diagnosis Observation Schedule [3]. Because of the resources and skill required to assemble and integrate this information, few centers offer ASD diagnostic evaluations, and these centers have lengthy waiting lists, ranging from 2–12 months for an initial appointment.Waiting is not only stressful for children with ASD and their families, but it delays their access to early intervention services, which have been shown to improve outcomes dramatically in many cases [4]. Furthermore, symptoms of ASD is very similar and can be easily confused with other mental illnesses whose treatment procedures are very different such as depression. To simplify the diagnostic process and shorten the waiting time, a computerized method for detecting ASD that requires little or no expert supervision would be a major advance over current practice.

We tested the feasibility and potential utility of a novel method for identifying children who may have ASD: natural language processing (NLP) with machine learning. The greatest challenges of researches on biomedical resources are the limitations on labeled data scale and data quality. First, it is not applicable to utilize crowd-sourcing tools for data labeling such as Amazon Mechanical Turk (AMT) because of privacy issues, which limits greatly on the data scale. Second, biomedical data for potential ASD patients are strictly restricted by privacy issues and the limited clinical resources for diagnosing ASD as there are only twelve ASD diagnosis centers in the USA. Moreover, the data resources which we have access to are complicated and noisy especially when the data includes hand-writing and even it is not stored in a usable format. For this initial study, we had access to the medical semi-structured and unstructured forms for 199 potential ASD patients in hand-written format. We converted all hand-written forms into digital format, extracted de-identified information from medical records obtained prior to the initial diagnostic evaluation, and examined whether our proposed algorithm could accurately predict which children should or should not receive an ASD diagnosis. Predictions are evaluated by an expert clinician in the Andrew J. Kirch Developmental Services Center at Golisano Childrens Hospital and confirmed by a standardized diagnostic instrument, the Autism Diagnostic Observation Schedule. To the best of our knowledge, our work is the first to propose a computerized ASD detection framework based only on hand-written semi-structured and unstructured medical forms. To be more specific, the results generated by our proposed framework with high recall values are suitable for identifying potential ASD patients who need to seek for further clinical help but shouldn’t be considered as a definite diagnosis. In particular, our contributions include the following:

- We propose a robust machine learning approach to tackle a challenging problem that involves mining from semi-structured and unstructured medical data in hand-written format.
- We convert semi-structured and unstructured medical forms into de-identified text contents in a ready-to-use format and same converting procedures can be used to extract information from confidential hand-written forms in large scales.
- We apply different word embedding models including the state-of-the-art distributed representations and establish a promising baseline for automated ASD detection on such a dataset.

## Related work

We are in an era of exploring data of all domains such as multimedia data from social networks, forms and videos from biomedical domain, and taking advantage of such data to benefit human lives. For example, researchers have used social multimedia data to monitor human’s mental health condition or emotion status. Other researchers have successfully recognized human sentiments based on recorded voice [5]. Yuan et al. [6] researched on analyzing users’ sentiment changes over time based on massive social multimedia data including texts and images from Twitter, and found strong correlation on sentiments expressed in textual contents and visual contents. Zhou et al. [7] integrated unobtrusive multimodal sensing such as head movement, breathing rate, and heart rate for mental health monitoring.

Much research has focused on medical applications and has involved machine-learning techniques. Compared with traditional biomedical diagnostic procedures which are usually time-consuming, labor-intensive, and limited to a small scope, new adoption of machine learning techniques into practical medical applications has advantages in terms of efficiency, scalability and reliability. For example, Devarakonda and Tsou developed a machine learning framework to automatically generate an open-end medical problem list for a patient using lexical and medical features extracted from a patient’s Electronic Medical Records [8]. Hernandez et al. [9] explored the feasibility of monitoring user’s physiological signals using Google glass and showed promising results. For diagnosing ASD, the most relevant data are observations of the child’s social communication and repetitive behavior. To obtain these data, we focus our research only on previously acquired records of potential patients, as these records contain comments about children’s behavior. The most similar work to ours is from [10], who analyzed digital early intervention records to detect ASD based on bigram and unigram features. Another research perspective on automated ASD assessment is to extract patterns from deficits in semantic and pragmatic expression [11, 12].

Another family of related work is on learning representations of texts, which embed words or documents into vector space of real numbers in a relatively low dimensional space such as [13]. Lexical features include *Bag-of-Words* (*BoW*), *n-grams* (typically bigram and trigram), and term frequency-inverse document frequency (*tf-idf*). Topic models such as Latent Dirichlet Allocation (*LDA*) are also used as features in document classification problems and researches show that topic model outperforms lexical features in some cases such as sentiment analysis [14, 15]. Recent word embedding algorithms are driven by the development of deep learning techniques. Distributed representations are obtained from a recurrent neural net language model [16, 17] by exploring the skip-gram model with subsampling of the frequent words and achieved a significant speedup and obtained more accurate representations of less frequent words.

## ASD detection framework

The biggest challenges in applying machine learning algorithms to medical studies are limited data scale, data labeling, and domain knowledge. Patients’ and non-patients’ data are more difficult to obtain compared with social media data due to the fact that fewer public biomedical data resources exist. For example, one video for medical use would require hours of recording and the participation of a doctor who has special expertise in such an area. These data are also kept strictly confidential unless patients expressly authorize release. In contrast, we can easily crawl thousands of tweets from Twitter about a certain topic in one hour. Additionally, data labeling and result evaluation would be another issue after data collection. Though crowd-sourcing techniques such as Amazon Mechanical Turk have been widely used for labeling in machine learning and computer vision tasks, they are not feasible for our case because we cannot reveal personal information to the crowd. We depend instead on reviews by expert clinicians for data labeling.

**Fig. 1:**
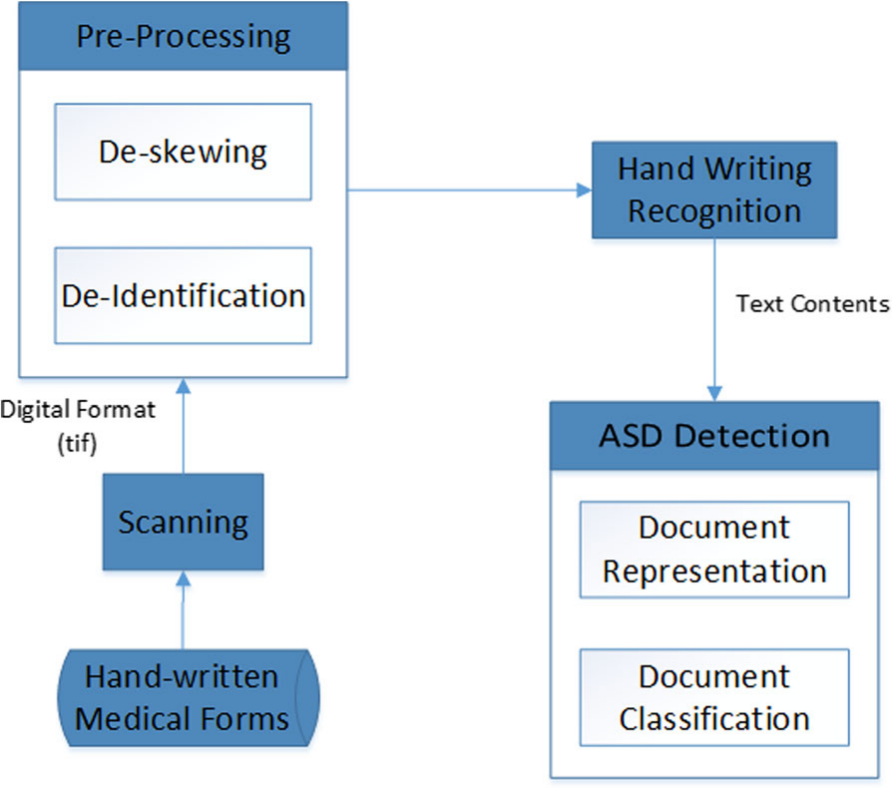
The framework of proposed ASD detection

In our case, we have collected hand-written medical forms from parents and service providers of children who have shown signs of ASD and thus need further rigorous evaluation. Those hand-written forms are far from a ready-to-use format since they are not even digitalized. Thus we first scan all the medical forms and save them in picture forms on a server that meets our institution’s stringent standards for maintaining confidentiality of electronic medical records and that is only accessible by authorized users for privacy concerns.We then conduct preprocessing procedures including de-skewing (meaning that we rotate the skewed scanned medical files to the right angle), and de-identification (automatically blanking areas containing personal information). OCR software is used to convert scanned documents into text contents. In the next step, we extract features based on the digital forms and perform classification using support vector machines to detect children with a high probability of having ASD. Features we extracted include lexical features such as *Bag-of-Words*, *n-gram* and term frequency-inverse document frequency (*tf-idf*), topic model (*LDA*) and distributed representation based on skip-gram model. Our proposed framework is shown in Fig. 1.

### Data collection

We have collected semi-structured and unstructured medical forms of children who have been referred for an evaluation of possible ASD. We first scan all the medical forms into digital format (tif) and go through preprocessing. In the next step, we incorporate the OCR software for recognizing text contents from the scanned documents. Hand-writing recognition is a well-established problem and we have experimented with different resources including Omnipage Capture SDK [18], Captricity [19], and ABBYY [20], which to the best of our knowledge are among the best tools in the market for recognizing hand-written letters and have been widely used in recognizing and transforming documents into usable digital forms [21]. Even so, the results are not satisfactory in some cases. We then inspect and manually correct data for all the medical documents that have been processed through OCR process in this case, which makes data collection much more time-consuming and labor-intensive.

In this study, we have digitized forms for 199 patients, with 56 children diagnosed as actual ASD patients (positive samples) and 143 non-ASD patients (negative samples). The medical forms we analyzed include: referral form from primary care physician, parent and teacher questionnaire, preschool and early intervention questionnaire, and additional forms including phone intake by social workers. All the forms for each potential patient are concatenated together and treated as one document for the classification. Ground truth labels of patients (ASD or not ASD) are obtained from clinical reports.

### Data preprocessing

A new problem arises by document scanning since sometimes the scanned forms are skewed. Additionally, the scanned forms contain personal information such as names, phone numbers, address and so on. Therefore, we go through preprocessing procedures including de-skewing, and de-identification. Such preprocessing procedure is necessary because OCR SDKs such as Captricity do not have embedded de-skewing option and their process involves recognizing documents slice by slice horizontally. Our preprocessing process improves the generated results significantly in most cases. By applying preprocessing, OCR process and manual correction afterwards, we are able to reduce the time of data collecting and converting by about 80%.

***De-skewing:*** We used a simple but effective de-skewing algorithm: first we compute entropy defined in Eq. 1 based on the probability that black pixel *x* appears in line *i* denoted by *P*_*α*_(*x*_*i*_) given a skew angle *α*, which is calculated as the count of black pixels in line *i* divided by the total number of pixels in the same line after skewed at angle *α*. We removed pixel lines which has less than 10% black pixels for two reasons: the value of function *P* *l* *o* *g*(*P*) rises with the increase on the value of *P* over range [ 0.1,1], but it acts the opposite way on range [ 0,0.1); and the *P*(*x*_*i*_) value of pixel lines containing text contents is usually larger than 10% expect for lines with pepper noise. We then find the optimized solution for *α* to minimize the entropy.

1 $$ H(X) = -\sum_{i}P_{\alpha }(x_{i})\log P_{\alpha }(x_{i})  $$

***De-identification:*** Since parts of medical forms are semi-structured, the regions containing personal information are located in relatively fixed areas for each type of form. Each form has its own distinctive feature such as edges in the parent’s questionnaire, which can be used to locate the areas needed to be de-identified. For unstructured forms, we manually black out the regions containing personal information. We use the following Sobel operator to extract edges of each medical form and automatically de-identify the information by blanking out such fields. We apply a pair of 3×3 convolution kernels as in Eq. 2.

2 $$ \begin{aligned} \left[ \begin{array}{ccc} -1 & 0 & 1\\ -2& 0 & 2\\ -1& 0 & 1 \end{array}\right] \left[ \begin{array}{ccc} 1 & 2 & 1\\ 0 & 0 & 0 \\ -1 & -2 & -1 \end{array}\right] \end{aligned}  $$

## Learning document representation and performing document classification

Learning good representations of documents to capture the semantics behind text contents is central to a wide range of NLP tasks such as sentiment analysis, and document classification as in our case.

### Lexical features

Lexical features are widely used in NLP tasks including *Bag-of-Words* model, *n-gram* model and *tf-idf*. These features capture the occurrences of words or phrases and usually contribute to high dimensional feature space of ten thousands depending on the dataset.

***Bag-of-Words and N-Gram Model:*** *Bag-of-Words* (*BoW*) model is a common way to represent documents in matrix form. A sentence or a document is represented as a vector of which the number of entities as the dictionary and each entity indicates the occurrence of that word in the input sentence or document. However, *BoW* model captures neither the ordering nor the semantic meanings of words. *N-gram* model is similar to *BoW* model with an extension from a bag of single words to a bag of, typically two-words or three-words phrases, know as bigram and trigram. *N-gram* model preserves ordering of the words and captures a better sense of semantics than *BoW* model.

***Term Frequency-Inverse Document Frequency:*** Both *BoW* and *n-gram* models draw much attention on frequent words with and without preserving the order of the words, which will be highly in favor of the frequent stop words such as: a/an, the, and, etc., and results in a noisy representation of the documents. While *Tf-idf* is considered as a weighted form of term frequency and is a statistical measure used to evaluate how important a word is to a document. Let *t* *f*(*w*,*d*) donate the number of times word *w* appears in document *d*, where document *d* belongs to a document set *D*, and *i* *d* *f*(*w*,*D*) indicates inverse document frequency of word *w* in the set *D*, then *tf-idf* is defined in Eqs. 3 and 4.

3 $$\begin{array}{@{}rcl@{}} tf-idf &=& tf(w,d) \times idf(w,D) \end{array} $$

4 $$\begin{array}{@{}rcl@{}} idf(w,D) &=& \log \frac{N}{1+\left | \left \{ d\in D:w\in d \right \} \right |} \end{array} $$

### Latent Dirichlet Allocation (*LDA*)

Assuming that each document is a mixture of latent topics, *LDA* is a probabilistic model which learns *P*(*t*|*w*), the probability of word *w* belongs to a certain latent topic *t* in a topic set *T* (usually with a pre-defined number of topics) [14]. By normalizing each word vector from a sentence or a document based on the probabilities of word-topic, we obtain the sentence or document vector for the topic distribution and thus embed the target document into a vector based on *LDA* model. Compared with lexical features mentioned above, the document representation learned by *LDA* model indicates the distribution of topics given the input word or document which is in a lower dimension and focusing more on the latent semantic meaning of the input texts.

### Distributed Representation (*Doc2vec*)

**Fig. 2:**
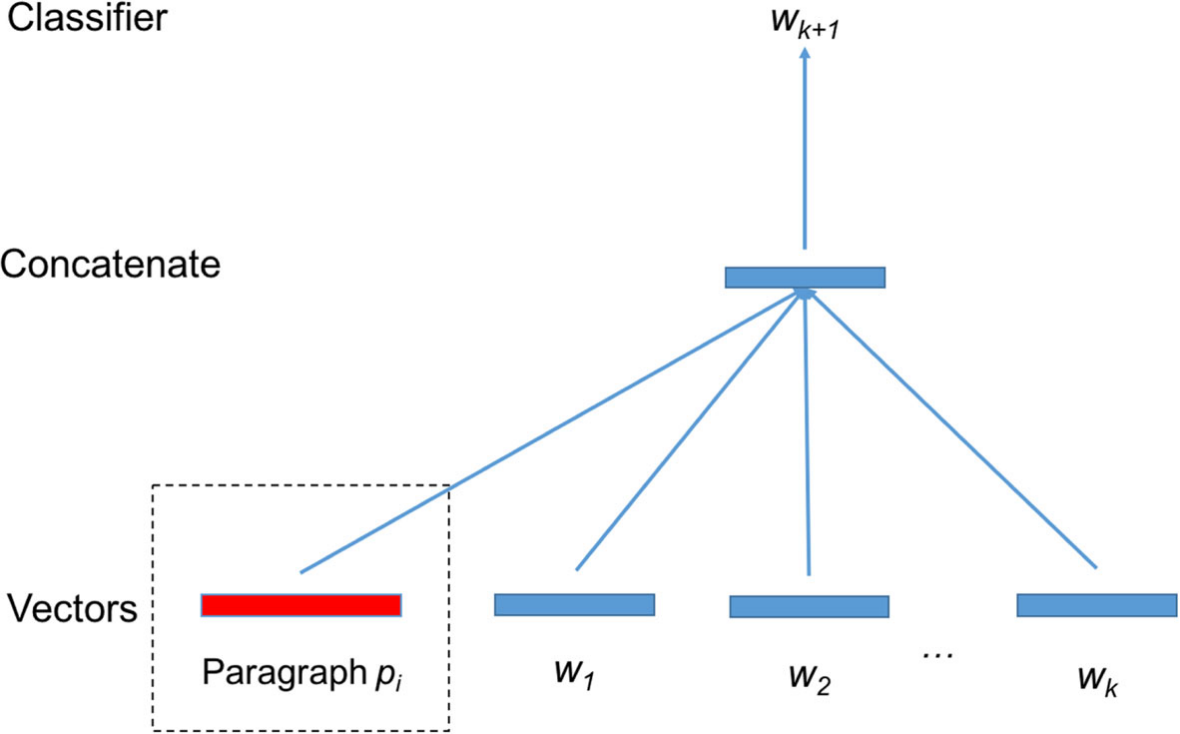
The framework of learning document representations

Following the work in [16, 17], we extracted the state-of-the-art distributed representations of the documents. Contrary to the lexical features, the semantic meaning conveyed by each word is assumed to distribute along a word window based on the distributed representations (as known as *doc2vec* feature) [17]. The *doc2vec* is learned based on the *word2vec* which can be trained in a Continuous Bag-of-Words (*CBoW*) or a Skip-gram fashion. For a word vector learning based on *CBoW* as shown in Fig. 2, given a sequence of *N* words {*w*_1_,*w*_2_,…,*w*_*N*_}, the objective is to predict the target word *w*_*i*_ given the surrounding words within a window size *c*:

5 $$ \frac{1}{N}\sum\limits_{i=c}^{N-c}\log p(w_{i}|w_{i-c},\ldots,w_{i+c})  $$

The probability of *w*_*i*_ in the objective function is calculated based on the softmax function shown in Eq. 6 where the word vectors are concatenated for predicting the next word in the content. The *Skip-gram* model is simply with the opposite direction of word prediction to the *CBoW* model where the objective is to predict the surrounding words given one word as input. Similarly, the processing of learning the *doc2vec* vector is maximizing the averaged log probability with the softmax function by combining the word vectors with the paragraph vector *p*_*i*_ in a concatenated fashion as shown in Fig. 2. In our case we choose to learn our document representations based on the *CBoW* model following the conclusions that it extracts better information when the data scale is limited and generally performs better in later classification tasks as demonstrated in [17].

6 $$ p\left(w_{i}|w_{i-c},\ldots,w_{i+c}\right) = \frac{e^{y_{w_{i}}}}{\sum_{j\in (1,\ldots,N)} e^{y_{wj}}}  $$

### Classification

***Upsampling:*** Since our dataset is imbalanced in that we have more negative samples, we upsample the positive samples before the training process. Our experimental results show an improvement over results without upsampling which will be discussed later in Section 5. For each pair of positive samples, we compute their Euclidean distance, and then find the nearest positive neighbours for each positive sample. Artificial positive samples are generated randomly between each positive sample and its nearest positive neighbours.

***Classifier:*** We use Support Vector Machines (SVM) for ASD detection. In order to extract discriminative features, we use lexical features, *LDA* model and *doc2vec* features. These features are useful, but they contribute to a relatively high dimensional space compared with our dataset scale. Such high dimensional spaces pose potential risk of overfitting and can reduce the robustness of our system. Therefore, when dealing with high dimensions, we add *L1*-regularization to our objective function to enforce the sparsity of weights as shown in Eq. 7. On the other hand, if the feature space is not in high dimensions, such as representations extracted from *LDA* and *doc2vec* model, we add *L2*-regularization term as shown in Eq. 8.

7 $$ \min_{w} \left \| w \right \|_{1} + C \sum\limits_{i=1}^{l}\left(\max \left(0,1-y_{i}w^{T}x_{i} \right)\right)^{2}  $$

8 $$ \min_{w} \frac{1}{2}w^{T}w + C\sum\limits_{i=1}^{l} \left(\max \left(0,1-y_{i}w^{T}x_{i} \right)\right)^{2}  $$

## Results and discussion

In this section, we demonstrate preprocessing results and evaluate our proposed ASD detection framework.

### Preprocessing Results

**Fig. 3:**
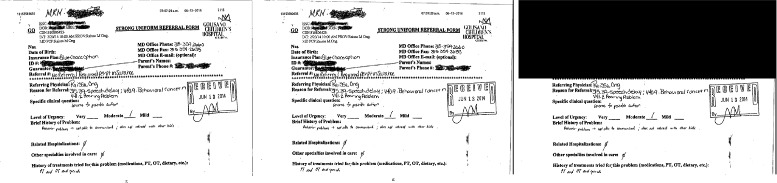
An example of semi-structured medical form (*left*), after de-skewing (*middle*) and de-identification (*right*)

Due to the page limit, we only show one example of a particular medical form (referral form from primary care physician) in Fig. 3 which is a semi-structured form. This form is clearly skewed to the left with a slight distortion which makes each line not straight, as demonstrated in Fig. 3 (left column). Such skewed documents will raise issues when passed to our OCR tools because the OCR tools will slice the document horizontally before text recognition and cut the words in the skewed lines in half. Our entropy-based de-skewing algorithm was able to find an optimal de-skewing angle and re-orient the form in a better shape. However, since the distortion exists, the computed optimal angle only assures the majority of lines and words to be horizontal as shown in the middle of Fig. 3. The top of this form contains confidential personal information which is kept above the line of asterisks including name, ID, phone number, etc. Our de-identification process tracked the line of asterisks automatically and blacked out the region above the line for as shown in Fig. 3 (right column). Our example in Fig. 3 is a semi-structured form and can be processed in an unsupervised manner, where we have the knowledge of document structure and can track the specific areas that include confidential information. For unstructured forms, personal information appears randomly on each form and it’s not feasible to recognize them all using algorithms with zero miss rate. Therefore we manually smeared the parts containing confidential information on each unstructured document and then passed the documents to the OCR tools to convert them into text contents, which makes our preprocessing semi-supervised in general.

### Classification results

Lexical features such as *BoW*, *tf-idf* and *n-gram* (we choose *bigram* and *trigram*) generated relatively high dimensional vector representations of target documents. We remove stop words (174 in total) such as “a”, “the”, “is/are”, “he/she”, etc. Our feature extraction results are shown in Table 1. We used Gensim to build *LDA* model and extract *doc2vec* features because its efficient implementation and good scalability [22]. We extracted 50, 100, 150, 200, 250, and 300 topics and features, respectively.

**Table 1:** Number of extracted lexical feature

	*BoW*	*TF-IDF*	*N-Gram*
Number of Features	4839	4839	9284

**Fig. 4:**
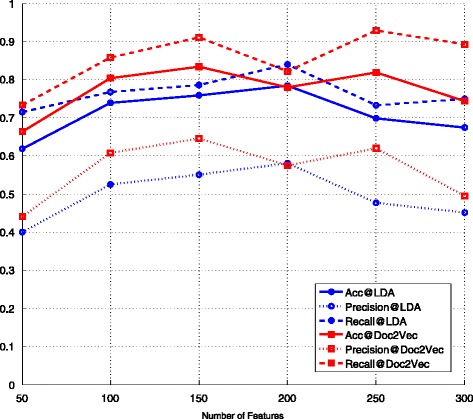
Classification results for *LDA* and *doc2vec* features with different dimensions

We used liblinear with *L1*-regularized and *L2*-regularized classification [23] for our document classification task. Since there are more negative samples in our dataset, we upsampled the positive samples before training. For lexical features, we chose to use *L1*-regularized SVM to reinforce the sparsity of feature space, and *L2*-regularized SVM for *LDA* and *doc2vec* features. Compared with the total 18962 dimensional feature space, only 386 weights learned for each feature are non-zero. We performed 7-fold cross-validation for evaluation and 5-fold cross-validation to learn the optimal parameters during the training process. For the training data of each fold, we generated two artificial positive samples for each positive sample which resulted in a more balanced dataset. Tables 2 and 3 and Fig. 4 show classification results including accuracy, precision and recall based on *BoW*, *tf-idf* and *n-gram*, and the combination of three, as well as *LDA* and *doc2vec* features. Since our application emphasizes on the recall over precision, the F2 scores are also provided (Eq. 9) in Tables 2 and 3. Given performances are different based on different numbers of features of *LDA* and *doc2vec*, we only show the best in Tables 2 and 3, which are 150 dimensions for *doc2vec* and 200 dimensions for *LDA* with upsampling, and 150 dimensions for *doc2vec* and 100 dimensions for *LDA* without upsampling.

9 $$ F2 = \frac{5\cdot precision\cdot recall}{4\cdot precision+ recall}  $$

**Table 2:** Classification results without upsampling

	Precision	Recall	F2 Score
*BoW*	33.2%	34.3%	34.1%
*Tf-Idf*	34.9%	36.7%	36.3%
*N-Gram*	36.7%	38.4%	38.1%
All Lexical Features	37.5%	46.2%	44.2%
*LDA*	39.7%	52.4%	49.2%
*Doc2Vec*	47.2%	64.4%	60.0%

**Table 3:** Classification results with upsampling

	Precision	Recall	F2 Score
*BoW*	40.4%	41.1%	40.9%
*Tf-Idf*	41.4%	42.9%	42.5%
*N-Gram*	43.1%	44.6%	44.3%
All Lexical Features	44.4%	42.9%	43.2%
*LDA*	58.0%	83.9%	77.0%
*Doc2Vec*	64.6%	91.1%	84.2%

As the results show that our proposed framework was toned towards a better performance on recall while maintaining a decent precision and accuracy because we don’t want to miss out any potential ASD patients. Comparisons between lexical features shows that the combination of all lexical features yields the best performance. Both *BoW* and *tf-idf* features perform similarly and *n-gram* features alone is very close to the combination of all three. On the other hand, features extracted using the *LDA* model show some improvements in precision and recall over all combinations of lexical features, but are neither significant nor as good as *doc2vec* features. The reason could be due to the fact that *LDA* emphasis on modeling topics from documents, and the use of *LDA* has no guarantee to generate robust document representations [15]. According to our experiment results, distributed representations provide the best classification results, and the best performance is obtained when the number of dimensions is 150. Additionally, as it is demonstrated in Fig. 4, more dimensions for *LDA* and *doc2vec* gain little improvements on the performance, if any. For the *LDA* model we expect there are not too many topics extracted from the data considering our data scale, and larger number of topics will render the effectiveness of the *LDA* model and add noise in the learned document vector. The reason for the *doc2vec* model is because the *doc2vec* model is expected to learn a decent semantic embedding of the documents within low dimensions, and in our case adding more dimensions will increase the risk of overfitting considering the scale of our dataset.

By applying upsampling, the precision and recall for *LDA* and *doc2vec* features raise significantly but only little improvements are obtained for lexical features. This is because generally *LDA* and *doc2vec* models learn a better representation of the documents, and the upsampling process we proposed enforces the positive samples’ representations while the data are well separated. On the other hand, the lexical features cannot learn features as effectively as *doc2vec* and for cases that positive and negative samples are not well separated such as lexical features, the proposed upsampling process doesn’t yield much improvements. The performance will benefit and become more robust when we provide a more balanced dataset. Table 4 demonstrates the top 10 features with the highest absolution weight values based on lexical features. These features are very consistent with clinical’s opinion on the keywords and key phrases regarding ASD diagnosis. However, the weights learned by the classifiers are not very distinguishable in value between each other, which shows that the document representations obtained by lexical features are not sufficient enough for a robust ASD detection.

**Table 4:** Top 10 selected features with the largest weights

Positive	Negative
Traits	Behavioral patterns
Seizures	Vocalizes vowel sounds
Attention span	Concerns
Physical	Actively involved
Disorder	Sure
Severely	Individual
Sensory	Help
Seems	Disability
Functionally plays	Affection family
Variety	Mood swings

## Conclusions

The reported prevalence of ASD has risen sharply over the past 25 years and the diagnosis of ASD is highly time-consuming and labor-intensive. Our proposed ASD detection algorithm has demonstrated high promise for detecting ASD based on the patients’ medical forms. Our method could significantly shorten the waiting time of the ASD diagnosis procedure and benefit the patients by facilitating potential early intervention services which have been proven to be very useful in many cases. Although the main focus of this paper is on ASD detection, the proposed NLP based framework can be potentially extended to other types of health related issues such as depression, anxiety, etc. For future work, we are working on computerized generation of an index for ASD patients indicating the severity of the patients based on their medical data, so that it can be used to monitor their progress over time. Furthermore, changes in the index could potentially serve as an outcome measure in trials of different therapies.
